# COST AND POLLUTANTS EMISSION REDUCTION WITH A TELEMEDICINE PROGRAM FOR HIP SURGERY IN BRAZIL

**DOI:** 10.1590/1413-785220253303e296058

**Published:** 2025-12-01

**Authors:** Fabio Seiji Mazzi Yamaguchi, Hector Fugihara Kroes, Gabriel Benevides Valiate Martins, Gustavo Estefan Lage, Vitor Matheus Silva, Henrique Melo de Campos Gurge

**Affiliations:** 1 Universidade de Sao Paulo, Faculdade de Medicina, Hospital das Clinicas, Instituto de Ortopedia e Traumatologia (HCFMUSP), Sao Paulo, SP, Brazil.; 2 Faculdade de Medicina da Universidade de Sao Paulo (FMUSP), Sao Paulo, SP, Brazil.

**Keywords:** Telemedicine, Hip Joint, Orthopedics, Air Pollution, Carbon Dioxide, Costs and Cost Analysis, Telemedicina, Quadril, Ortopedia, Poluição do Ar, Dióxido de Carbono, Custos e Análise de Custo

## Abstract

**Objective:**

To evaluate the economic and environmental impacts of telemedicine use and the correlation of socioeconomic variables with telemedicine preference in patients with hip pathologies at a tertiary referral center in São Paulo, Brazil.

**Methods:**

A cross-sectional study (January-June 2024) analyzed telemedicine patients, collecting data on preferences, socioeconomic profile, travel, and costs (transport and food). Avoided distance, time, and pollutant emissions were calculated using Google Maps and emission factors. The preference for telemedicine was correlated with socioeconomic data. Statistical analyses used Wilcoxon, chi-square, and logistic regression tests.

**Results:**

148 patients were included, of whom 77.7% preferred telemedicine. The mean round-trip distance avoided was 168.84 km, and the mean time saved was 223.97 minutes. Estimated out-of-pocket savings were USD 12.62 for public transport users and USD 28.95 for private car users. Telemedicine also reduced emissions by approximately seven metric tons of carbon dioxide in total. Higher income was positively associated with telemedicine preference (p=0.0283); other variables showed no significant associations.

**Conclusion:**

Telemedicine reduced time, costs, and emissions, improving access. Preference was higher among wealthier patients, indicating barriers for low-income groups. Further studies should explore low adherence among socioeconomically disadvantaged populations. *Level of Evidence IV; Economic and Decision Analysis.*

## RESUMO

**Objetivo:**

Avaliar os impactos econômicos e ambientais da telemedicina e a correlação de variáveis socioeconômicas com a preferência por telemedicina em pacientes com patologias do quadril atendidos em centro de referência terciário em São Paulo, Brasil.

**Métodos:**

Estudo transversal (janeiro a junho de 2024) com pacientes atendidos por telemedicina, com dados sobre preferências, perfil socioeconômico, deslocamento e custos (transporte/alimentação). Distância, tempo e emissões evitadas foram estimados com o Google Maps e fatores de emissão. Correlacionou-se a preferência por telemedicina com dados socioeconômicos. Utilizaram-se testes de Wilcoxon, qui-quadrado e regressão logística.

**Resultados:**

Participaram 148 pacientes, dos quais 77,7% preferiram a telemedicina. A média de distância de deslocamento evitada foi de 168,84 km, com economia média de tempo de 223,97 minutos. Pacientes que utilizavam transporte público e carro particular economizaram, respectivamente, US$ 12,62 e US$ 28,95. A telemedicina também reduziu as emissões de poluentes, evitando aproximadamente sete toneladas de dióxido de carbono. A preferência pela telemedicina esteve positivamente associada à maior renda (p = 0,0283). As demais variáveis não apresentaram associações estatisticamente significativas.

**Conclusão:**

A telemedicina reduziu o tempo, custos e as emissões, melhorando o acesso. A preferência foi maior entre pacientes com maior renda, indicando barreiras para os grupos de baixa renda. Novos estudos devem explorar a baixa adesão entre populações em situação de vulnerabilidade socioeconômica. *Nível de Evidência IV; Análises econômicas e de decisão.*

## INTRODUCTION

Telemedicine delivers healthcare services remotely using technologies such as videoconferencing and phone calls. This approach enables consultations, diagnoses, and monitoring without in-person visits.^1^ Following the onset of the COVID-19 pandemic in March 2020, many healthcare services adopted telemedicine programs as an alternative to in-person consultations, ensuring continuity of care and significantly increasing its use.^2^

Numerous studies have analyzed the socioeconomic impacts of telemedicine, highlighting its expansion of patient access to healthcare services, particularly where distance and travel time are significant barriers.^3-5^ Furthermore, research has demonstrated the potential of this approach to mitigate environmental impacts by reducing pollutant emissions from fuel combustion during patient travel.^6,7^ Such studies are particularly relevant to Brazil, considering the large regional and sociodemographic disparities in access to healthcare, as well as the adverse effects of pollutant emissions on public health and, consequently, on the country's public health system.^8-11^

This study evaluates the socioeconomic and environmental impact of an orthopedic telemedicine program for Hip Group patients at a tertiary referral hospital in São Paulo. Launched in 2021, the program primarily serves residents of the capital but also includes patients from other municipalities and states. We compare travel distance, time, and out-of-pocket costs, together with transport-related emissions, including greenhouse gases and air pollutants, between telemedicine visits and hypothetical in-person consultations at the same hospital.

## METHODS

This cross-sectional study was conducted with patients receiving care via telemedicine. Ethical approval was obtained from the Research Ethics Committee of the Hospital das Clínicas, Faculty of Medicine, University of São Paulo (approval number 5.022.929; CAAE: 51877521.0.0000.0068). Patients provided consent through a digital informed consent form (ICF). During virtual consultations, participants were invited to join the study by completing a structured questionnaire. The questionnaire collected data on demographic characteristics, place of residence, modes of transportation used for in-person consultations, the need for companions, and expenses related to transportation and meals.

### Population

The study included patients who attended telemedicine outpatient consultations provided by the Hip Group between January and June 2024. Exclusion criteria included individuals who did not sign the informed consent form, those who failed to respond to all questions in the questionnaire, and participants who withdrew from the study at any stage.

### Distance and travel time

The distance and travel time between the patients’ residences and the hospital in São Paulo were calculated using Google Maps, considering the fastest route. Travel time was estimated based on the mode of transportation indicated by the patient, with a fixed arrival time of 8:00 AM. The same route was used to estimate both distance and travel time. Final values were doubled to account for the round trip.

### Cost calculation

Transportation and meal costs were calculated based on the round-trip journey from the patient's residence to the hospital. For individuals using a private car, fuel consumption was estimated at 10 km/L, with the price of gasoline set at BRL 5.74 per liter as of March 2024.^12-13^ For public transportation, costs were based on round-trip ticket prices according to Google Maps. Additionally, meal expenses were estimated at BRL 15.00 for unaccompanied patients and BRL 30.00 for those accompanied. All costs were presented in USD and calculated with the purchasing power parity exchange rate of BRL 2.3 per USD as per CCEMG–EPPI Centre Cost Converter, designed to facilitate international comparison of costs.^14^

### Pollutant emissions

For the calculation of emissions, travel distances were multiplied by standardized emission factors for "Passenger Cars and Light Commercial Vehicles" and "Urban and Intercity Buses," as published by Diana Maria Cancelli and Nelson Luís Dias. To estimate the reduction in emissions, only the pollutants generated by fuel combustion were considered, assuming the patient had opted for an in-person consultation.^12^

### Statistical analysis and figure generation

The relationship between continuous variables and the preference for telemedicine was analyzed using the Shapiro-Wilk test for normality. Depending on the normality, either the Student's t-test or the Wilcoxon test was applied to continuous variables. Logistic regression was used to explore significant relationships, and the chi-square test was employed to evaluate associations with categorical variables. Mean and standard deviation were used for descriptive statistics (significance level: 0.05). The Clipcoords v0.1.0 software^15^ was used for geocoding, and all analyses were performed using R v4.3.1 (RStudio v2023.09.1).

## RESULTS

Data were collected from 150 patients, of whom 2 were excluded for using modes of transportation not covered by the study, resulting in 148 eligible participants. Of these, 3 were further excluded from the analyses of distance, travel time, and pollutant emissions due to geocoding failures.

Among the 148 eligible participants, 115 (77.7%) preferred telemedicine, while 33 (22.3%) preferred in-person consultations (Table 1).

**Table 1:** Data on travel to IOT-HCFMUSP categorized by preferred consultation modality.

Variables	Telemedicine N = 115	In-person consultation N = 33	Total
**Round-trip distance saved (km)**			
Mean (SD)	179.45 (498.83)	132.48(214.06)	168.84 (450.22)
Median (IQR)	62.00 (40.00, 120.00)	58.00 (40.00, 90.00)	62.00 (40.00, 118.50)
Range (min, max)	4.00; 5,046.00	12.00; 818.00	4.00; 5,046.00
Sum	20,278.00	4,372.00	24,650.00
**Round-trip time saved (min)**			
Mean (SD)	230.46 (408.62)	201.76 (165.34)	223.97 (367.63)
Median (IQR)	160.00 (122.00, 244.00)	146.00 (120.00, 174.00)	156.00 (120.50, 216.50)
Range (min, max)	20.00; 4,320.00	50.00, 752.00	20.00; 4,320.00
Sum	26,042.00	6,658.00	32,700.00
**Means of Transportation**			
Private Car	64 (56%)	14 (42%)	78 (53%)
Public Transport	51 (44%)	19 (58%)	70 (47%)
**Patients' Place of Origin**			
City of São Paulo	45 (40%)	17 (52%)	62 (43%)
Other Cities within the State of São Paulo	61 (54%)	14 (42%)	75 (52%)
Out of State	6 (5.4%)	2 (6.1%)	8 (5.5%)

**Figure 1:**
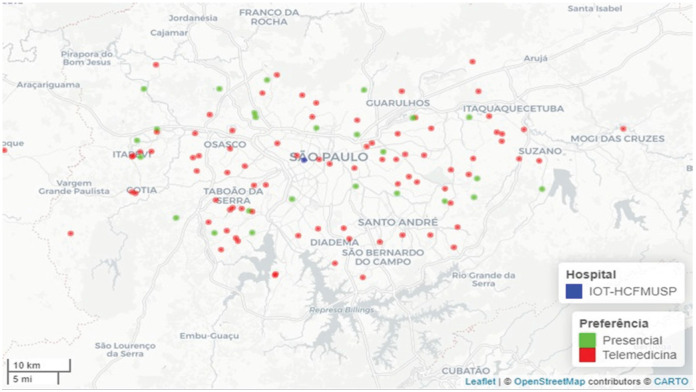
Geographic distribution of patients served by the orthopedic telemedicine program, covering the city of São Paulo.

Source: Authors’ elaboration based on data collected in the IOT-HCFMUSP telemedicine program (2022–2023); Basemap: © OpenStreetMap contributors; Tiles: © CARTO; Rendered With: Leaflet.

**Figure 2:**
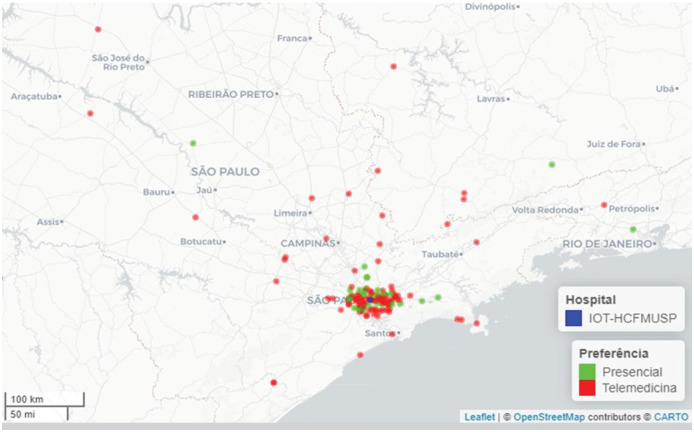
Geographic distribution of patients served by the orthopedic telemedicine program, covering the state of São Paulo.

Source: Authors’ elaboration based on data collected in the IOT-HCFMUSP telemedicine program (2022–2023); Basemap: © OpenStreetMap contributors; Tiles: © CARTO; Rendered With: Leaflet.

**Figure 3:**
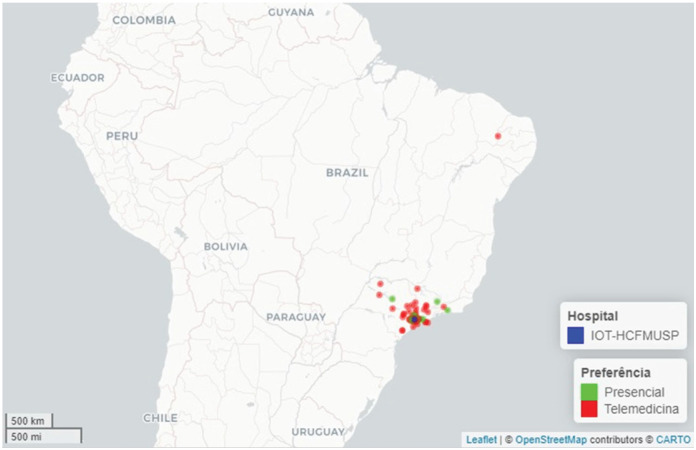
Geographic distribution of patients served by the orthopedic telemedicine program, covering all regions of Brazil.

Source: Authors’ elaboration based on data collected in the IOT-HCFMUSP telemedicine program (2022–2023); Basemap: © OpenStreetMap contributors; Tiles: © CARTO; Rendered With: Leaflet.

Most of the patients lived either in the municipality of São Paulo (43%) or other municipalities within the state (52%), and only a minority (5.5%) in other states (Figures 1-3). The median distance for all patients would be 62 km (IQR: 40–118.50 km), with a minimum of 4 km and a maximum of 5,046 km. This distance would be travelled with a median duration of 156 minutes (IQR: 120.50–216.50), with durations ranging from 20 to 4,320 minutes. The distribution of this data is present in Figure 1. To travel, 78 (53%) would use private cars, and 70 (47%) would rely on public transportation (Table 1).

The median income found was 2.00 (IQR 1.00–3.00) brazilian minimum wages (BRL 1,412.00 / USD 613.91). The median cost associated with attending in-person consultations was USD 13.47 (IQR 10.32-21.59), with a median cost-to-income ratio of 1.53% (IQR 0.67–2.93). Additionally, 72 patients (49%) required an accompanying person. Patient absence from work was necessary for 51 patients (34%), while 36 patients (24%) also indicated their accompanying person would need to miss work (Table 2).

**Table 2:** Socioeconomic elements divided by consultation modality preference.

Variables	Telemedicine N = 115	In-person consultation N = 33	Total
**Income (minimum wages)**			
Mean (SD)	2.10 (1.08)	1.78 (1.22)	2.03 (1.12)
Median (IQR)	2.00 (1.00, 3.00)	1.00 (1.00, 2.00)	2.00 (1.00, 3.00)
Range (min, max)	0.00, 6.00	0.80, 5.00	0.00, 6.00
**Cost**			
Mean (SD)	48.66 (49.06)	49.51 (57.21)	48.85 (50.80)
Median (IQR)	30.84 (23.80, 55.54)	31.65 (23.80, 47.60)	31.07 (23.80, 49.80)
Range (min, max)	4.02, 303.22	0.00, 264.77	0.00, 303.22
Sum	5,547.43	1,633.77	7,181.20
**Cost/Income (%)**			
Mean (SD)	2.31 (2.97)	3.11 (4.51)	2.49 (3.38)
Median (IQR)	1.45 (0.60, 2.66)	1.80 (1.15, 3.14)	1.53 (0.67, 2.93)
Range (min, max)	0.11, 18.06	0.00, 20.06	0.00, 20.06
**Meal-related expenses**			
Yes	84 (73%)	27 (82%)	111 (75%)
No	31 (27%)	6 (18%)	37 (25%)
**Presence of companion**			
Yes	55 (48%)	17 (52%)	72 (49%)
No	60 (52%)	16 (48%)	76 (51%)
**Patient work absenteeism**			
Yes	44 (38%)	7 (21%)	51 (34%)
No	71 (62%)	26 (79%)	97 (66%)
**Companion work absenteeism**			
Yes	28 (24%)	8 (24%)	36 (24%)
No	87 (76%)	25 (76%)	112 (76%)

Data analysis revealed a positive and statistically significant association between patients’ income and their preference for online consultations (p = 0.0283), although the logistic regression model used did not reach statistical significance (coefficient = 0.2840; p = 0.1531). The mean income in the group favouring telemedicine was 2.10 ± 1.08 minimum wages, compared to 1.78 ± 1.22 minimum wages in the group preferring in-person consultations.

While the average distance and travel time for the telemedicine group (179.45 ± 498.83 km and 230.46 ± 408.62 minutes) were higher than those for the in-person consultation group (132.48 ± 214.06 km and 201.76 ± 165.34 minutes), no significant relationship was observed between these variables and the preference for consultation type. Similarly, no significant associations were found with municipality of residence, cost, use of public transportation, presence of a companion, or the need for the patient or companion to take time off work.

During data collection regarding transportation for a hypothetical in-person consultation, some patients were unable to reliably report information such as fares, fuel consumption, or parking costs. Therefore, it was only possible to calculate environmental impact estimates for 119 of the 148 participants. Patients who would have used public buses (n = 42) avoided the emission of a median of 24.92 kg of carbon dioxide (CO_2_), 28.0 g of nitrogen oxides (NO_*x*_), 504.0 g of non-methane hydrocarbons, and 11.2 g of particulate matter (PM) per consultation. Similarly, for patients using private vehicles and with successfully calculated distances (n = 77), telemedicine prevented the release of 13.44 kg of CO_2_, 25.6 g of NO_*x*_, 0.64 g of aldehydes, 25.6 g of non-methane hydrocarbons, 0.96 g of PM, and 9.6 g of methane (CH_4_) per consultation (Table 3).

**Table 3:** Emissions avoided through the implementation of a telemedicine consultation strategy, divided by means of Transportation.

Variables	Public Transport N = 42	Private Car N = 77	Total
**Carbon dioxide (kg)**			
Mean (SD)	105.25 (351.87)	35.18 (43.30)	59.91 (212.97)
Median (IQR)	24.92 (15.13, 36.05)	13.44 (9.66, 48.72)	17.80 (10.71, 38.72)
Range (min, max)	3.56; 2,245.47	2.94, 199.92	2.94; 2,245.47
Sum	4,420.63	2,709.00	7,129.63
**Nitrogen oxides (g)**			
Mean (SD)	118.26 (395.36)	67.01 (82.47)	85.10 (243.51)
Median (IQR)	28.00 (17.00, 40.50)	25.60 (19.40, 92.80)	25.60 (17.30, 55.60)
Range (min, max)	4.00; 2,523.00	5.60, 380.80	4.00; 2,523.00
Sum	4,967.00	5,160.00	10,127.00
**Non-methane hydrocarbons (g)**			
Mean (SD)	2,128.71 (7,116.43)	67.01 (82.47)	794.67 (4,310.44)
Median (IQR)	504.00 (306.00, 729.00)	25.60 (18.40, 92.80)	107.20 (22.40, 325.60)
Range (min, max)	72.00; 45,414.00	5.60, 380.80	5.60; 45,414.00
Sum	89,406.00	5,160.00	94,566.00
**Particulate matter (g)**			
Mean (SD)	47.30 (158.14)	2.51 (3.09)	18.32 (95.70)
Median (IQR)	11.20 (6.80, 16.20)	0.96 (0.69, 3.48)	3.03 (0.84, 8.37)
Range (min, max)	1.60; 1,009.20	0.21, 14.28	0.21; 1,009.20
Sum	1,986.80	193.50	2,180.30
**Methane (g)**			
Mean (SD)	-	25.13 (30.93)	25.13 (30.93)
Median (IQR)	-	9.60 (6.90, 34.80)	9.60 (6.90, 34.80)
Range (min, max)	-	2.10, 142.80	2.10, 142.80
Sum	-	1,935.00	1,935.00
**Aldehydes (g)**			
Mean (SD)	-	1.68 (2.06)	1.68 (2.06)
Median (IQR)	-	0.64 (0.46, 2.32)	0.64 (0.46, 2.32)
Range (min, max)	-	0.14, 9.52	0.14, 9.52
Sum	-	129.00	129.00

## DISCUSSION

No national studies were identified that would allow a direct comparison with our findings. However, when analyzing international studies and adjusting values based on Purchasing Power Parity (PPP) to U.S. dollars (USD),^14^ the following data were observed: in the U.S., Dullet NW et al. reported average savings of 447 km, 245 minutes, and USD 195.^16^ In a Colombian population, Prada SI et al. documented an average reduction of 73 km, 108 minutes, USD 10 in fuel, and USD 17.5 in public transportation costs.^17^ In Mali, Bagayoko CO et al. reported approximate savings of USD 50 in transportation costs.^18^ Direct comparison between populations with such distinct socioeconomic profiles is complex. While Dullet et al. reported significant savings, their study focused on a rural population in California, where patients travelled proportionally longer distances than observed in other studies, including the present one.^16^ While Prada et al. reported the smallest savings, reflecting a Colombian population that mostly resided within the same city as the hospital, when considering subpopulations outside the "Valle del Cauca" region, the observed savings align more closely with our study.^17^ Lastly, although Bagayoko CO did not detail distances, times, or criteria used to calculate transportation costs, the reported values are similar to those identified in our analysis.^18^ Similarly to what Bagayoko highlighted in his article, in Brazil—a country with significant income inequality—such expenditures represent a substantial burden for a large portion of the population, with the costs of a single in-person consultation amounting to up to 20% of a patient's monthly income.

Barriers to healthcare access are a widely discussed topic in the literature. Among these barriers, hospital distance and access to transportation are particularly relevant, especially in vulnerable populations disproportionately burdened by diseases.^19,20^ These difficulties may result in poor adherence to medical appointments, increased hospitalizations, and worse management of chronic conditions. National studies addressing the Brazilian context support these trends, highlighting the need for measures to mitigate these barriers.^8,9^ In this context, telemedicine emerges as a promising alternative, enabling patients with access difficulties to receive high-quality remote care. However, our analysis identified a significant positive correlation between income and preference for telemedicine, which raises questions, as an inverse correlation was expected, given that low-income populations face greater access barriers. This underscores the complexity of factors influencing healthcare access, highlighting potential barriers for lower-income populations. Levesque JF et al. describe five essential capacities to effectively interact with healthcare services: perceiving service availability, willingness to use it, having access, affording the costs, and engaging.^21^ To better explore these dimensions in the telemedicine context, a more comprehensive analysis of socioeconomic factors affecting usability is needed.

From the perspective of pollutant emission reductions, the fuel savings for the 119 patients with a single online consultation equate to the monthly per capita emissions of approximately 39 Brazilians.^22^ During data collection regarding transportation for a hypothetical in-person consultation, some patients were unable to reliably report information such as fares, fuel consumption, or parking costs. Therefore, it was only possible to calculate environmental impact estimates for 119 of the 148 participants. Furthermore, there was a significant reduction in emissions of nitrogen oxides (NOx), non-methane hydrocarbons, particulate matter, and aldehydes—pollutants directly associated with fossil fuel combustion, known to exacerbate respiratory and cardiovascular diseases. Reducing these pollutants contributes not only to better air quality but also to fewer hospitalizations and lower mortality, positively impacting public health and reducing healthcare costs.^6,7,23^

Reducing greenhouse gas (GHG) emissions, such as carbon dioxide, nitrogen oxides, and methane, directly contributes to mitigating climate change. Less GHG in the atmosphere means reduced global warming and its consequent impacts, such as rising sea levels, extreme weather events, and ecosystem changes. The decrease in particulate matter improves air quality, reducing respiratory and cardiovascular problems among the population. The reduction in non-methane hydrocarbons also enhances air quality and decreases photochemical smog, minimizing health and environmental impacts.^24^

Limitations of the current study include the limitation on the information about the transportation methods used by the patients in the event of an in-person consultation. The methodology employed in this study included a standard emission measure that considers only the vehicle category, as specific details about individual fuel types for each vehicle were not available. While this approach provides a generalized estimation of avoided emissions, it highlights the need for improved data collection methods in future studies to achieve greater specificity. This limitation is an inherent challenge in studies analyzing the environmental impacts of telemedicine, but it does not detract from the clear benefits demonstrated by this analysis.

Additionally, the lack of detailed information on the usability of telemedicine as perceived by patients may introduce confounding factors, complicating the interpretation of our findings. Administering validated questionnaires and evaluating their usability could help clarify these factors.

## CONCLUSION

This study demonstrated that orthopedic Telemedicine in Brazil provides significant economic and environmental benefits. The adoption of this modality substantially reduced patient travel time and costs, while also contributing to a decrease in pollutant emissions generated by the combustion of fossil fuels. This reduction in emissions highlights telemedicine's potential to improve health access, mitigate the effects of socioeconomic inequalities and climate change and its impacts on public health, particularly in São Paulo, where improved air quality may help reduce expenses related to respiratory and cardiovascular diseases. However, additional research is necessary to better understand the barriers and preferences associated with telemedicine use, especially among low-income populations.
